# Reversible Human TGF-β Signal Shifting between Tumor Suppression and Fibro-Carcinogenesis: Implications of Smad Phospho-Isoforms for Hepatic Epithelial-Mesenchymal Transitions

**DOI:** 10.3390/jcm5010007

**Published:** 2016-01-12

**Authors:** Katsunori Yoshida, Miki Murata, Takashi Yamaguchi, Koichi Matsuzaki, Kazuichi Okazaki

**Affiliations:** Department of Gastroenterology and Hepatology, Kansai Medical University, 2-5-1, Shin-machi, Hirakata, Osaka 573-1010, Japan; muratami@takii.kmu.ac.jp (M.M.); yamaguct@hirakata.kmu.ac.jp (T.Y.); matsuzak@takii.kmu.ac.jp (K.M.); okazaki@hirakata.kmu.ac.jp (K.O.)

**Keywords:** epithelial-mesenchymal transition (EMT), hepatic stellate cells (HSC), liver fibro-carcinogenesis, myofibroblast (MFB), Smad, transforming growth factor-β (TGF-β)

## Abstract

Epithelial-mesenchymal transition (EMT) and mesenchymal-epithelial transition (MET) are observed during both physiological liver wound healing and the pathological fibrotic/carcinogenic (fibro-carcinogenetic) process. TGF-β and pro-inflammatory cytokine are considered to be the major factors accelerating liver fibrosis and promoting liver carcinogenesis. Smads, consisting of intermediate linker regions connecting Mad homology domains, act as the intracellular mediators of the TGF-β signal transduction pathway. As the TGF-β receptors, c-Jun *N*-terminal kinase and cyclin-dependent kinase, differentially phosphorylate Smad2/3, we have generated numerous antibodies against linker (L) and *C*-terminal (C) phosphorylation sites in Smad2/3 and identified four types of phosphorylated forms: cytostatic COOH-terminally-phosphorylated Smad3 (pSmad3C), mitogenic pSmad3L (Ser-213) signaling, fibrogenic pSmad2L (Ser-245/250/255)/C signaling and migratory pSmad2/3L (Thr-220/179)/C signaling. After acute liver injury, TGF-β upregulates pSmad3C signaling and terminates pSmad3L (Ser-213)-mediated hepatocyte proliferation. TGF-β and pro-inflammatory cytokines cooperatively enhance collagen synthesis by upregulating pSmad2L (Thr-220)/C and pSmad3L (Thr-179)/C pathways in activated hepatic stellate cells. During chronic liver injuries, hepatocytes persistently affected by TGF-β and pro-inflammatory cytokines eventually become pre-neoplastic hepatocytes. Both myofibroblasts and pre-neoplastic hepatocyte exhibit the same carcinogenic (mitogenic) pSmad3L (Ser-213) and fibrogenic pSmad2L (Ser-245/250/255)/C signaling, with acquisition of fibro-carcinogenic properties and increasing risk of hepatocellular carcinoma (HCC). Firstly, we review phospho-Smad-isoform signalings in epithelial and mesenchymal cells in physiological and pathological conditions and then consider Smad linker phosphorylation as a potential target for pathological EMT during human fibro-carcinogenesis, because human Smad phospho-isoform signals can reverse from fibro-carcinogenesis to tumor-suppression in a process of MET after therapy.

## 1. Introduction

An epithelial-mesenchymal transition (EMT), firstly reported by Elizabeth Hay in 1982, is a biologic process that allows a polarized epithelial cell, which normally undergoes multiple biochemical changes, to be able to assume a mesenchymal cell phenotype [1]. The EMT is believed to play an important role in tissue fibrosis [2]. Undergoing an EMT refers to the loss of apicobasal polarity in epithelial cells; intercellular adhesion complexes undergo dramatic phenotypic change, causing them to become nonpolar and, thus, allowing these cells to move through the ECM like mesenchymal cells [2]. The components of EMT include enhanced migratory capacity, invasiveness, elevated resistance to apoptosis and greatly increased production of extracellular matrix (ECM). EMT has been classified into three different biological subtypes based on the biological context. Type 1 EMT occurs during the embryonic stage and produces primary mesenchymal cells. Type 2 EMT is associated with wound healing, tissue regeneration and organ fibrosis. Type 3 EMT is observed in carcinoma cells, and promotes tumor invasion and metastasis [3]. A number of growth factors and cytokines that regulate liver EMT; among them, transforming growth factor (TGF)-β is the most potent factor engaged in order to initiate and reach completion of EMT [4].

TGF-β enhances hepatic stellate cell (HSC) activation, stimulates collagen gene transcription and suppresses matrix metalloproteinases (MMP) expression. Thus, TGF-β, as well as its intracellular mediators, Smad proteins, can be potential therapeutic targets for liver fibrosis. TGF-β inhibits hepatocyte proliferation, but it also promotes hepatocellular carcinoma (HCC). TGF-β has been shown to play both tumor-suppressive and tumor-promoting roles [5,6,7]. As disease progresses toward malignancy, HCC gains advantage by selective reduction of the tumor-suppressive activity of TGF-β together with augmentation of TGF-β oncogenic activity [6]. In concert with mitogens, such as pro-inflammatory cytokines and growth factors, TGF-β induces the accumulation of extracellular matrix (ECM), while mitogenic signaling antagonizes cytostatic TGF-β function [8]. Recent studies have emphasized the possibility of the Smad family’s involvement in the pathogenesis of fibrosis and carcinogenesis (fibro-carcinogenesis) [9].

Current evidence suggests that regulation of ECM accumulation by fibrogenic TGF-β signals involves different mechanisms in acute and chronic liver injuries, although plasma TGF-β is elevated and thought to control liver regeneration and fibrosis in both situations [10,11]. After acute liver injury, most hepatic cells rapidly enter the cell cycle and complete liver regeneration without excessive deposition of ECM, which results in tissue dysfunction [12,13,14]. On the other hand, as a result of chronic liver damage, HSC undergo progressive activation to become myofibroblast (MFB)-like cells, leading to cirrhosis, which is characterized by the formation of regenerative nodules in liver parenchyma separated by fibrotic septa [15,16]. Several conditions in chronically-damaged livers favor human hepatocarcinogenesis, mostly resulting from recurrent cycles of cellular proliferation, inflammation and fibrosis. Within this anomalous environment, certain clones of hepatocytes acquire proliferative and survival advantages, eventually forming dysplastic nodules, the histological substrate of HCC [17].

Unraveling the molecular mechanisms of EMT signal in a patho-physiologic condition is critical to our understanding of its role in disease and the development of its therapies [18]. In this review, we first imply the TGF-β signal in the presence of pro-inflammatory cytokines for hepatic EMT. We next show the similarities and differences of the TGF-β signal between epithelial and mesenchymal cells in physiological and pathological conditions. We then consider the TGF-β signal as a potential target for pathological EMT during human fibro-carcinogenesis, because the human TGF-β signal can reverse from fibro-carcinogenesis to tumor-suppression in a process of mesenchymal-to-epithelial transition (MET) after therapy.

## 2. Signaling Pathways Controlling Liver EMT

### 2.1. Hepatic EMT

Type 1 EMT in the liver is associated with implantation, embryo formation and organ development. It is also characterized by a program organized to generate diverse cell types that share common mesenchymal phenotypes [19]. In the fetal liver, hepatocytes are detected in various intermediate stages of EMT (defined as cells that express both mesenchymal and hepatocyte markers), further suggesting that at least during fetal development, hepatocytes can contribute to the emergence of cells with mesenchymal features. In turn, type 1 EMT can generate the primary mesenchymal cells that have the potential to subsequently undergo a MET to generate a secondary epithelial structure. MET occurs in physiological (development) and pathological situations (cancer metastasis), where migrating mesenchymal-like cells that have reached secondary sites reacquire cell-cell contacts and polarity [20]. Because the MET represents a reversion of EMT, a downregulation of EMT-inducing transcription factors, such as Snail and Slug, is invariably associated with MET. Although MET molecular mechanisms are only partially characterized, it is suggested that TGF-β and bone morphogenetic protein (BMP)-dependent signaling can promote MET [21].

Organ fibrosis can be classified as type 2 EMT, which is associated with tissue repair and involves secondary epithelial or endothelial cells transitioning to resident tissue fibroblasts in response to persistent inflammation. Type 2 EMT can continue to respond to ongoing inflammation and lead to the expression mesenchymal markers on cells, which can advance to various extents through an EMT. TGF-β downregulates epithelial and hepatic markers, such as E-cadherin and albumin, and gains mesenchymal markers, such as vimentin, α-smooth muscle actin (α-SMA) and β-catenin [22,23,24,25]. If the cells ultimately shed all of their epithelial markers and gain a complete fibroblastic phonotype, the cells have undergone a complete EMT. The partial EMT refers to an intermediate phenotype as the cell transition, with progressive loss of epithelial markers and acquiring mesenchymal markers [26]. The accumulating evidence has suggested that the EMT contributes to liver fibrosis and carcinogenesis [27,28].

Type 3 EMT occurs in neoplastic cells that have previously undergone genetic and epigenetic changes. As the disease progresses toward advanced stages, EMT plays an important role in liver fibrosis and tumor progression. In this context, EMT is elicited by several oncogenic pathways, such as Ras, Wnt/beta-catenin and TGF-β [29]. In particular, Ras-mitogen-activated protein kinase (MAPK) has been shown to activate two related transcriptional factors known as Snail and Slug [30]. Both of these proteins are transcriptional repressors of E-cadherin, and their expression induces EMT [31]. Snail expression, in fact, significantly increases along with HCC differentiation, accelerating cancer invasion. EMT and E-cadherin downregulation have been shown to play an important role in HCC progression. Other relevant pathways in EMT observed in the metastatic include the transcription factor Twist and FOXC2, an important player during embryonic development [32,33], as well as the involvement of microRNAs [33].

A number of studies demonstrated that TGF-β induces EMT in freshly-isolated mouse hepatocyte [23,27,34]. Zeisberg *et al.* reported that hepatocyte EMT was observed in CCl_4_-induced liver fibrosis, and they also demonstrated that the inhibition of the TGF-β pathway limited the extent of liver fibrosis by a cell fate tracing technique [35]. These results suggest that TGF-β is the most established mediator and regulator molecule in liver EMT and fibrosis. Within the inflammatory microenvironment, TGF-β is secreted by platelets and Kupffer cells. A significant increase in TGF-β expression is observed in the activated HSC, thus indicating that TGF-β acts as an autocrine positive regulator for liver EMT and resulting in fibrosis.

Taura *et al.* reported extremely fascinating data. They examined the expression of epithelial and mesenchymal markers by a cell lineage strategy in mice. Hepatocytes isolated from these transgenic mice were able to undergo EMT in culture when incubated with TGF-β. However, in mice chronically treated with CCl_4_, no cells exhibited a double labeling specific for both hepatocytes and collagen-expressing cells [36]. Moreover, many studies failed to define EMT rigorously or to differentiate between the transition to a mesenchymal *vs*. a myofibroblast phenotype [37,38]. These observations suggest that hepatocytes may not undergo EMT *in vivo*. However, more recently, Michelotti *et al.* have reported that HSC could differentiate into hepatocytes during many types of liver injury [39]. This evidence suggests that it is premature to conclude whether EMT occurs in human liver injury. Future studies using more improved lineage tracing techniques are indispensable for the resolution of this controversial issue.

During the past decade, EMT has been increasingly recognized to occur during HCC progression. The escape of carcinoma cells from the solid tumor might be due to the dedifferentiation of epithelial cells, which occurs by loss of cell-to-cell contacts and the concomitant gain of migratory and invasive abilities. This phenotypical conversion of HCC cells plays a pivotal role in the dissemination of malignant hepatocytes during HCC progression.

### 2.2. Involvement of Both Pro-Inflammatory Cytokines and TGF-β Signals via JNK in Hepatic EMT

After acute liver damage, hepatocytes regeneration and ECM deposition cooperatively restore the original liver. As a result of chronic liver insults, excessive scar response and epithelial proliferation lead to liver fibro-cirrhosis. Plasma TGF-β increases after both acute and chronic liver inflammation and plays an important role in EMT processes.

Many signaling pathways, including TGF-β, epidermal growth factor (EGF) and platelet-derived growth factor (PDGF), and oncogenic events, such as Ras activation, are implicated in EMT induction, both in physiology and pathology [3,40]. In particular, TGF-β is considered the master EMT inducer for malignant and non-malignant epithelial cells, including hepatocytes [4]. TGF-β acts as a potent inducer of EMT, combining both Smad-dependent and -independent signaling pathways [41]. Reviewing TGF-β signaling is essential for better understanding of the liver EMT.

c-Jun *N*-terminal kinase (JNK) is a serine/threonine kinase affecting proliferation, differentiation, survival and migration. In JNK1^−/−^ mice, both fibrosis and HCC development are prevented. Collagen deposition is marked in wild-type and JNK2^−/−^ mice, but is less dense in JNK1^−/−^ mice, suggesting the importance of JNK1 in the development of liver fibrosis [42]. JNK1^−/−^ mice exhibit impaired liver carcinogenesis, with smaller and fewer tumor masses [43]. Importantly, JNK1^−/−^ mice displayed decreased HCC proliferation in a carcinogenic model and decreased hepatocytic growth in a model of liver regeneration. In both instances, impaired proliferation is caused by increased expression of p21^WAF1^, a cell-cycle inhibitor, and reduced expression of c-Myc, a negative regulator of p21^WAF1^.

## 3. Multiple Phospho-Isoforms of Smad2 and Smad3 Exist

### 3.1. TGF-β Signaling

Progress over the past 20 years has disclosed important details of how the TGF-β family propagates its signaling [44,45,46,47]. The Smads make up a group of intracellular proteins that have the critical role of transmitting TGF-β signals to the nucleus. The Smads are categorized into three subgroups, the receptor-activated Smads (R-Smads), the common Smads and the inhibitory Smads. In cell-signaling pathways, various transcription factors are phosphorylated at multiple sites by upstream kinases. Catalytically-active TGF-β type I receptor (TβRI) phosphorylates COOH-tail serine residues of R-Smad, which include Smad2 and the highly-conserved protein Smad3, with 91% identity in amino acid sequence [45,46]. Both proteins have two conserved domains: Mad homology (MH)1 and MH2 and less conserved linker regions, which separate the two domains. The MH1 domain is responsible for DNA binding, and the MH2 domain binds to transcription co-activators and co-repressors. Mitogenic signals alternatively cause phosphorylation of R-Smad at specific sites in their middle linker regions [48,49,50,51,52]. After, phosphorylated R-Smad rapidly oligomerizes with common Smad, Smad4. This complex translocates to the nucleus, where it regulates the transcription of target genes. Smad7 is an inhibitory Smad that is expressed in response to a prolonged TGF-β signal [53,54].

Our recent analysis showed that pro-inflammatory cytokines simultaneously activate linker-phosphorylated Smad2/3 [51,55,56]. To elucidate how the pro-inflammatory cytokines modulate TGF-β signaling through Smad2/3 linker phosphorylation, we generated several types of antibodies (Abs), which selectively react with phosphorylated Smad2/3. Abs reactive with structurally-related phosphorylated peptides are emerging as valuable tools for determining phosphorylation sites *in vivo* and for investigating their distinct signals via phosphorylated domains. Domain-specific phospho-Smad2/3 Abs have allowed us to reveal that TβRI and JNK differentially phosphorylate Smad2 and Smad3 to create three phosphorylated forms (phosphoisoforms): COOH-terminally-phosphorylated Smad (pSmad2C and pSmad3C), linker phosphorylated Smad (pSmad2L and pSmad3L) and dually-phosphorylated Smad (pSmad2L/C and pSmad3L/C) [51,57,58,59]. Except for pSmad2L with cytoplasmic localization [46,58], the other phospho-isoforms are localized to cell nuclei [50,51,55,56,60,61,62,63,64]. TGF-β mediated pSmad3C signaling represents a major growth inhibitory signal in normal epithelial cells, such as hepatocytes [5]. In the context of cell cycle control, the most important targets of action by TGF-β are the genes encoding the two CDK inhibitors p15^INK4B^ and p21^Cip1^ that inhibit CDKs and downregulate c-Myc expression [65].

### 3.2. JNK Signaling

Proinflammatory cytokines use non-Smad signaling pathways, including the JNK and p38 MAPK pathways, to convey the same fibrogenic signals [66]. The non-Smad pathway is generally considered an important effecter via the Ras pathway [67,68], promoting cell proliferation, invasion and fibrosis. TGF-β also uses non-Smad signaling pathways and promotes fibrosis and tumorigenesis. TGF-β and pro-inflammatory cytokines elicit signaling responses through JNK/non-Smad pathway [66]. Tumor necrosis factor (TNF) receptor-associated factor 6 (TRAF6) and TGF-β-associated kinase 1 (TAK1) have recently been shown to be crucial for the activation of the MAPK [69,70,71]. The TAK1 pathway is known to regulate cell survival, migration and invasion. Especially important among genes induced by the JNK pathway are the two immediate early genes encoding the Fos and c-Jun transcription factors. Once synthesized, these proteins can associate with one another to form activator protein (AP)-1, a widely-acting heterodimeric transcription factor that is often found in hepatocarcinogenesis and liver fibrosis [72].

### 3.3. Smad Phospho-Isoform Signaling

TGF-β and JNK pathways act as potent inducers of EMT, combining both Smad-dependent and -independent signaling pathways [41]. Although TGF-β canonical Smad and non-Smad signaling pathways at first appear to diverge from each other, there are indications that they often converge toward each other; for instance, tumor suppressive TGF-β signaling is suppressed during human carcinogenesis. We consider Smad phospho-isoform signaling pathways and learn how C-tail phosphorylation antagonistically or synergistically acts with linker phosphorylation to transmit a cytostatic or pro-tumorigenic TGF-β signal. We further reported how the linker phosphorylation-mediated non-canonical Smad pathway promotes fibro-carcinogenesis and tumor progression. Our concepts can elucidate many aberrant phenomena that could not be outlined by the canonical Smad pathway and the non-Smad pathway. Using our domain-specific anti-phospho-Smad antibodies, we have categorized Smad phospho-isoform signaling into four classes: cytostatic pSmad3C signaling; mitogenic pSmad3L (Ser-213) signaling; fibrogenic pSmad2L (Ser-245/250/255)/C; and migratory pSmad2/3L (Thr-220/179)/C signaling [8]. PSmad3L (Ser-213) antagonizes Smad3 *C*-terminal phosphorylation at the cell membrane [9,50,56], whereas pSmad2L (Ser-245/250/255) or pSmad2/3L (Thr-220/179) transmits the fibrogenic/pro-tumorigenic TGF-β signal after their C-tails’ phosphorylation [50,51,56,73].

Numerous reports have suggested that TGF-β enhances invasion and metastasis by switching TGF-β signaling from the canonical Smad pathway to the pro-tumorigenic/fibrogenic non-Smad pathway [67,68]. However, in cancer cells, Smad signaling indeed drives pro-tumorigenic gene expression [66,74] and tumor-initiating cell stemness [75]. Linker phosphorylation-mediated Smad signaling can elucidate long-standing controversial questions, because linker phosphorylation occurs in non-canonical Smad signaling, bringing about cell growth, invasion and fibrosis via the Ras/MAPK and CDK pathways [49,50,54,55,75]. TGF-β/JNK can activate both the Smad pathway through linker phosphorylation and the non-Smad pathway [75], usually operating in parallel [73]. These observations indicate that Smad signaling, through linker phosphorylation governed by Ras, could be controlled by and functions in conjunction with the alternative non-Smad pathway [67,68]. Collectively, linker phosphorylation-mediated Smad signaling will be recognized as a major non-canonical Smad pathway.

## 4. Involvement of Smad Singaling in EMT

### 4.1. Mitogenic pSmad3L (Ser-213) Pathway

JNK can phosphorylate Smad3 at the linker region [76]. In contrast to cytoplasmic retention of pSmad2L (Ser-245/250/255), pSmad3L (Ser-213) is not retained in the cytoplasm. Both pSmad3C and pSmad3L (Ser-213) can form hetero-complexes with Smad4, and the Smad complex moves to the nucleus [56]. Because nuclear hetero-oligomerization is essential to the assembly of target-specific transcriptional complexes [77], Smad3 can utilize two different phospho-domains to transmit different signals, as both a tumor suppressor and a tumor promoter [8].

Linker phosphorylation can modify COOH-terminally-phosphorylated Smad2/3 signaling [48,49,50,51,56,78]. JNK-mediated pSmad3L and TβRI-mediated pSmad3C signals oppose each other; most importantly, the balance can shift between cell growth and growth inhibition. Ser-213 phosphorylation of Smad3 indirectly inhibits its COOH-terminal phosphorylation and subsequently suppresses tumor-suppressive pSmad3C signaling. By using genetic, as well as pharmacologic approaches, we showed that blockade of linker phosphorylation abolished oncogenic properties in Ras-transformed cells and restored the TβRI/pSmad3C-mediated tumor-suppressive function present in parental epithelial cells [50].

### 4.2. Fibrogenic TβRI/JNK/pSmad2L (Ser-245/250/255)/C Signaling

Activated JNK retains most Smad2 proteins in the cytoplasm [48,50]. Smad2 can accumulate in the nucleus only if its C-terminus is phosphorylated under conditions of sustained linker phosphorylation by JNK. Smad2- or Smad3-deficient mouse embryo-derived fibroblasts suggest that both Smad2 and Smad3 are required for the induction of the plasminogen activator inhibitor (PAI)-1 [79]. Smad3 and Smad4 cooperatively activate the PAI-1 promoter in a TGF-β-independent manner [80]. The Smad3 mutant (Smad3SD), in which the *C*-terminal serines are replaced by aspartic acids, is localized in the nucleus to activate PAI-1 transcription in a TGF-β-independent fashion [81]. Importantly, the Smad3SD mutant lacks the induction of target genes required for growth inhibition [81]. Moreover, the Smad3 phospho-mimetic mutation in the linker domain enhances PAI-1 mRNA and protein [82]. pSmad2L (Ser-245/250/255)/C undergoes translocation to the nucleus, where it binds to pSmad3L (Ser-213) and the Smad4 complex [51,55], which in turn stimulates PAI-1 transcription [55]. PAI-1 facilitates cell invasion [83] and induces ECM deposition [84]. These observations suggest cross-talk between TGF-β, and proinflammatory cytokine-induced non-Smad signaling and the non-canonical Smad pathway in the nucleus appear to play important roles during the liver fibrosis and carcinogenesis. The recognition of non-Smad and the non-canonical Smad pathway as a potent driver of fibro-carcinogenesis makes it urgent to investigate in more detail the molecular mechanisms by which TGF-β promotes its fibro-carcinogenic effects.

### 4.3. Immature pSmad2/3L (Thr-220/179)/C Signaling

pSmad2L (Thr-220)/C and pSmad3L (Thr-179)/C signals play important roles in the maintenance of stem cells [85]. During colonic epithelial wound repair, Wnt5a potentiates TGF-β-dependent pSmad3C signaling in colonic stem cells to promote crypt regeneration [86]. Moreover, pSmad2/3L (Thr-220/179)/C signaling has been reported to be a specific marker for stem cells for human and murine stomachs, intestines and colonic crypts [87,88]. These findings suggest that colonic stem cells responding to TGF-β and other mitogens released by stromal cells might proliferate beyond the bottom of the crypts and migrate upward, directed by pSmad2L (Thr-220)/C- and Smad3L (Thr-179)/C signaling. Better understanding of the immature pSmad2/3L (Thr-220/179)/C signaling in the maintenance of stem cell phenotype is an important area for future investigation.

## 5. Similarities and Differences of the TGF-β Signal between Epithelial and Mesenchymal Cells in Physiological and Pathological Conditions

### 5.1. Acute Liver Injury (Physiological Type 2 EMT)

After partial hepatectomy and chemical damage, most hepatic cells rapidly enter the cell cycle and undergo an average of approximately 1.6 cycles of replication per cell to completely restore the original liver mass. This physiological process is orchestrated by the interplay of cytokine and growth factor [12]. TNF, hepatocyte growth factor (HGF) and the complement have been identified and recognized as playing important roles in regenerating the liver. TNF binds its receptor and activates nuclear factor kappa B in Kupffer cells, which produce IL-6 and TNF. IL-6 initiates hepatocyte proliferation by activation of STAT-3.

Plasma TGF-β levels also increased after acute liver injury, and the anti-proliferative response to TGF-β decreased in hepatocytes by downregulation of TGF-β receptor expression in rat livers [10,11]. In HSC, whenever TGF-β is increased, TGF-β could transduce its signal for ECM production via its receptor, because signaling receptors were expressed constantly [11].

**Figure 1:**
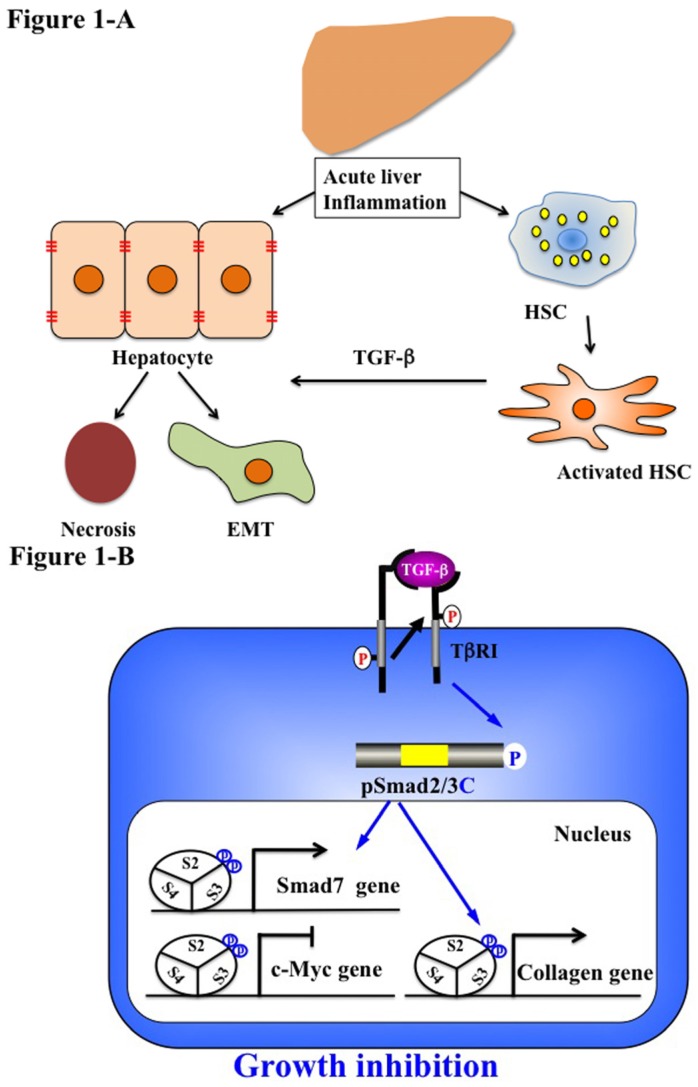
Liver regeneration-related EMT and phospho-Smad signaling in acute liver disease. (**A**) Quiescent hepatic stellate cells (HSC) are characterized by retinoid droplets in the cytoplasm. Acute liver injury caused HSC activation and hepatocyte damage, necrosis and EMT. Activated HSC move from the space of Disse to sites of damage where the activated HSC contribute to tissue repair by producing large amounts of collagen. HSC also play an important role in secreting TGF-β. (**B**) Catalytically-active TβRI phosphorylates COOH-tail serine residues of Smad2 and Smad3. Both pSmad2C and pSmad3C are localized in the nuclei of hepatocytes and mesenchymal cells in acute injured liver. After binding with Smad4, pSmad2/3C translocate with Smad4 to the nucleus and bind to the collagen promoter. pSmad2/3C stimulate extracellular matrix (ECM) deposition and suppress cell growth by c-Myc inhibition. However, Smad7 induced by the pSmad3L/C signal terminates the fibrogenic phospho-Smad signaling. This negative feedback mechanism of the fibrogenic TGF-β/CK signal results in a transient collagen synthesis in the activated HSC, which may thus contribute to tissue repair.

Although the majority of injured hepatocytes undergo apoptosis, profibrotic growth factors and TGF-β can induce hepatocytes to undergo phenotypic and functional changes of the partial or typical type of EMT in rat models (Figure 1A) [89]. Activation of intrahepatic stem cells, such as hepatocyte progenitor cells, oval stem cells and bone marrow stem cells, is the main source of the exceptional regenerative capacity. From the available evidence, examples of acute liver hepatitis are followed by complete or near-complete resolution and return of the liver to normal [90].

Numerous types of molecules, including TGF-β, monocyte chemoattractant protein (MCP) 1 and TNF by inflammatory cells and resident activated HSC, cause disruption of the epithelial layers via degradation of the basement membrane. We further examined in more detail TGF-β signaling in hepatocytes and HSC during acute liver injury, focusing on pSmad2L/C and pSmad3L/C pathways in chemically-injured rat livers [55,63]. These phospho-isoforms are involved in collagen synthesis and transmit a proliferative, invasive TGF-β signal in mesenchymal cells [55,63]. Nuclear localization of pSmad2L/C and pSmad3L/C is seen in the activated HSC [63]. In particular, strong Smad2/3 phosphorylation at the COOH-tail and threonine residues in the linker regions is observed in the activated HSC (unpublished data). Because TGF-β, pro-inflammatory cytokines and PDGF activate the JNK pathway in HSC [63], pro-inflammatory cytokines and PDGF can convert a cytostatic TGF-β signal into a collagen-producing character in activated HSC under the influence of inflammatory microenvironments (Figure 1B). Collectively, pSmad2L/C and pSmad3L/C signaling may mobilize HSC from the space of Disse to sites of damage, where the activated HSC contribute to tissue repair by producing large amounts of collagen.

In HSC after acute liver injury, TβRI activated by endogenous TGF-β signal phosphorylated Smad3C further upregulating Smad7 transcription [91]. Subsequently, Smad7 terminates fibrogenic signals mediated by pSmad2L/C and pSmad3L/C and could be involved in the transient response to the autocrine TGF-β signal after acute liver injury [76,91]. In the same way, the activation of Smad2/3 was tightly restricted in primary cultured HSC [76,90]. Taken together, Smad7 is involved in this tight restriction of the non-canonical Smad signaling in HSC and regulates the intensity and duration of the TGF-β responses [92].

### 5.2. Chronic Liver Injury (Pathophysiological Type 2 EMT)

**Figure 2:**
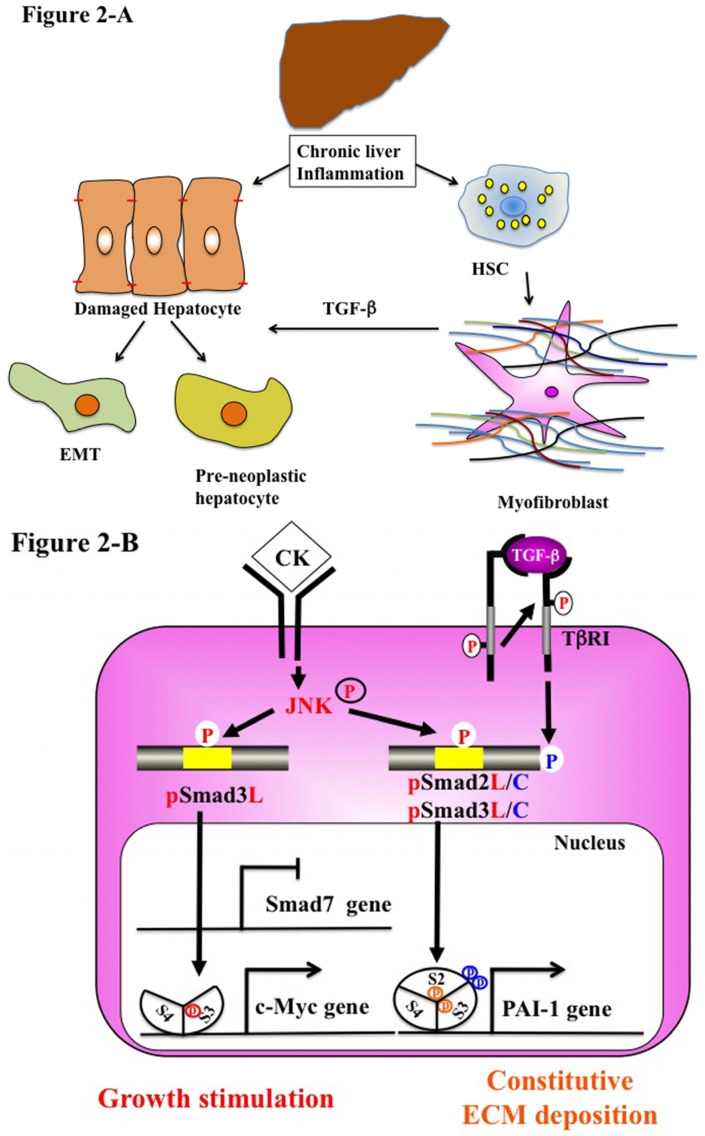
Liver fibro-carcinogenesis-related EMT and phospho-Smad signaling during chronic liver disease. (**A**) Prolonged exposure to chronic injury; HSC undergo constitutive activation to become myofibroblasts (MFB)-like cells, which persistently induce deposition of ECM and liver fibrosis. Continuous insults will shift EMT-like cells to complete EMT and pre-neoplastic hepatocytes. (**B**) During chronic liver injury, pro-inflammatory cytokines (CK), such as TNF-α activate JNK, result in phosphorylation of both Smad2L and Smad3L, both in MFB and pre-neoplastic hepatocyte. P-Smad3L translocates with Smad4 to the nucleus and binds the PAI-1 promoter. After COOH-tail phosphorylation of cytoplasmic pSmad2L by TβRI, pSmad2L/C translocates to the nucleus. Both pSmad2L/C and pSmad3L stimulates PAI-1 transcription and ECM deposition, while they suppress the pSmad3C-mediated tumor suppressive pathway. Pre-neoplastic hepatocytes exhibit the same oncogenic (mitogenic) pSmad3L and fibrogenic pSmad2L signaling as MFB, thereby accelerating liver fibrosis and increasing the risk of HCC. In contrast to Smad7 induction in HSC via the pSmad3C pathway, pSmad3L cannot induce Smad7 in MFB and pre-neoplastic hepatocytes (left). Under a low level of Smad7, the fibrogenic phospho-Smad signaling can constitutively promote ECM deposition by MFB, which may eventually develop into accelerated liver fibro-carcinogenesis.

Chronic inflammation causes progressive liver fibrosis (Figure 2A). Fibrogenesis is a mechanism of wound healing and repair [16]. However, prolonged injury causes deregulation of the normal processes and results in extensive deposition of ECM proteins and fibrosis [93]. Following liver injury of any etiology, HSC undergo activation. Activated HSC show increased proliferation, motility and ECM production [94,95]. A number of cytokines, continuously released by damaged Kupffer cells and endothelial cells, can change activated HSC to MFB [96]. These include TGF-β, PDGF and endothelin-1, which stimulate transcription factors, such as Sp1, c-Jun, STAT-1 and Smad proteins, that regulate gene expression [97,98,99,100]. MFB perpetuate their own activation through several autocrine loops, including the secretion of TGF-β and upregulation of its receptors [91].

Following chronic liver injury, there is a marked accumulation of α-SMA and vimentin-positive cells at the sites of active liver fibrosis [101,102]. α-SMA was detected in fibrotic human and rat livers around fibrotic septa, which indicates the presence of transition hepatocytes. Furthermore, EMT was also reported in cirrhotic liver cells derived from murine CCl_4_-induced models. Interestingly, *in vitro* TGF-β treatment induced higher vimentin expression in cirrhotic liver-derived hepatocytes than in normal liver-derived hepatocytes. Cells isolated from cirrhotic livers can exhibit anti-apoptosis effects in contrast to normal hepatocytes under TGF-β treatment. This evidence suggests EMT-like cells, even EMT cells, exist during chronic liver injury and gain mesenchymal features. Therefore, chronic inflammation promotes pathological type 2 injury in the liver.

MFB are fully stimulated via autocrine TGF-β signaling and display a strong intrinsic R-Smad activation. During transdifferentiation from HSC to MFB in culture, the pSmad3C-mediated signal decreases while the pSmad3L (Ser-213) pathway predominates [55]. These observations fully support the finding of pSmad3L (Ser-213) rather than pSmad3C in nuclei of α-SMA-immunoreactive MFB in portal tracts of chronically-HCV-infected liver specimens [62]. In contrast to a transient increase in Smad7 in the activated HSC after acute liver injury, Smad7 remained at a low level in MFB throughout chronic liver injury. The lack of Smad7 induction as observed in MFB in chronic liver disease could be one reason for excessive TGF-β effects during the progression of liver fibrosis [91,103].

Convincing evidence has shown that TGF-β can induce EMT by activation of the Snail transcription factor, which is a key molecule in the EMT, and repression of epithelial markers, such as E-cadherin in murine hepatocytes [22,23,24,25]. E-cadherin expression levels vary dramatically in different human tumors, and an inverse relationship between levels of E-cadherin and patient survival has been documented [104]. In this regard, mutations in the E-cadherin gene have been identified in cancer cells, making them more susceptible to EMT and metastasis [105,106]. A high level of TGF-β in HCC patients can induce EMT and promote HCC progression and metastasis [107].

Fibrosis also promotes carcinogenesis [108,109]. During chronic liver injury, activated MFB secrete large amounts of ECM proteins. Hepatocytes are replaced with abundant ECM, mainly in the form of fibrillar collagen. Affected hepatocytes also participate in liver fibrogenesis by stimulating deposition of ECM proteins. Similar to MFB, hepatocytes in chronic injured livers exhibit Ser-213 phosphorylation at Smad3L, particularly those adjacent to inflamed portal tracts [62]. Thus, hepatocytes are regulated by the same pSmad3L (Ser-213) pathway as are MFB (Figure 2B). The extent of the phosphorylation at Smad3L (Ser-213) is less in hepatocytes distant from portal tracts, in sharp contrast to pSmad3C, which is predominantly located in hepatocytic nucleus distant from portal tracts [62]. TGF-β and pro-inflammatory cytokines are released from infiltrating Kupffer cells in portal tracts to activate JNK [76,110]. These finding suggest that pro-inflammatory cytokine-dependent JNK can convert Smad3 to pSmad3L (Ser-213) in both affected hepatocytes and MFB in chronic hepatitis. These data also demonstrate that type 2 EMT promotes phenotypic change at the premalignant phase in chronic injured hepatocytes.

Studies suggest that MFB promote tumor progression. MFB have been found at the invasive fronts of tumors where they secrete pro-invasive cytokines, proteases and inflammatory mediators [111]. Fibrotic lesion and MFB have also been found in the tumor microenvironment prior to cancer cell invasion into the stroma, suggesting that MFB may mediate an invasive phenotype [112]. Activation of an EMT program has been proposed as the critical mechanism for the acquisition of an invasive phenotype by epithelial cancer cells. Dooley *et al.* have reported that Smad7 inhibits pSmad2C-mediated signaling and reduced TGF-β-mediated fibrogenesis [103]. Moreover, IFN-γ exerts antifibrotic effects by upregulation of Smad7 [113]. We have examined that the pSmad2L (Ser-245/250/255)/C pathway transmits fibrogenic signals by stimulating PAI-1 transcription with pSmad3L (Ser-213). Thus, TGF-β and pro-inflammatory cytokines can mediate pSmad2L/C and pSmad3L signaling, which induce PAI-1 expression and promote ECM deposition in hepatocytes and MFB, accelerating fibrosis.

### 5.3. Stem Cells and Advanced HCC Biology (Type 3 EMT)

Stem cells, which have unlimited and long life time potential, are the basis for tissue homeostasis in the adult organism. TGF-β signals have been reported to play important roles in the maintenance of the self-renewal and pluripotency of stem cells. Increasing evidence suggests that tumors develop and progress from a small subset of cells with the ability to self-renew and produce non-stem differentiated cells. Liver stem cells, existing quiescently within the canals of Hering in adults, are activated for compensative proliferation and differentiation into both hepatic and biliary lineages [114]. It is reported that HCC are derived from liver cancer stem cells, which are mostly transformed from normal stem cells [115,116]. Recently, Wu and Fan have reported that chronic TGF-β stimulation promotes EMT and gives rise to cancer stem cells in HCC [117,118]. TGF-β-induced EMT promotes cancer stem cell properties and higher invasive capability [117]. Fernando *et al.* have proven that CD44-positive HCC cells exhibit features of EMT and showed increased chemoresistant potential [119]. Therefore, TGF-β induced EMT signaling may play important roles in the generation of high-grade invasive and chemo-resistant cells with stem cell-like features in HCC.

## 6. Reversible Human TGF-β Signal Shifting between Tumor-Suppression and Fibro-Carcinogenesis

JNK increases the basal phosphorylation of Smad3L and downregulates TGF-β-dependent cytostatic actions of pSmad3C in hepatocytes during chronic hepatic injuries. Inhibition of JNK/pSmad3L (Ser-213) signaling could decrease carcinogenic signaling. Pharmacologic interference to restore the lost tumor-suppressive function in chronic liver disorders could be a key therapeutic aim for the prevention of hepatic carcinogenesis [120]. Nagata *et al.* administered a JNK inhibitor SP600125 to rats and succeeded to suppress chemical carcinogenesis by shifting hepatocytic Smad3 signaling from the carcinogenic pSmad3L (Ser-213) pathway to the tumor-suppressive pSmad3C pathway [64]. This evidence provides that JNK/pSmad3L (Ser-213) is an important target for the development of chemopreventive and therapeutic measures to reduce the emergence of HCC in the context of chronic liver injury and to slow the progression of existing tumors. Further studies, including JNK inhibitors, are necessary for new molecular targeting therapy for better improvement of HCC prognosis.

**Figure 3:**
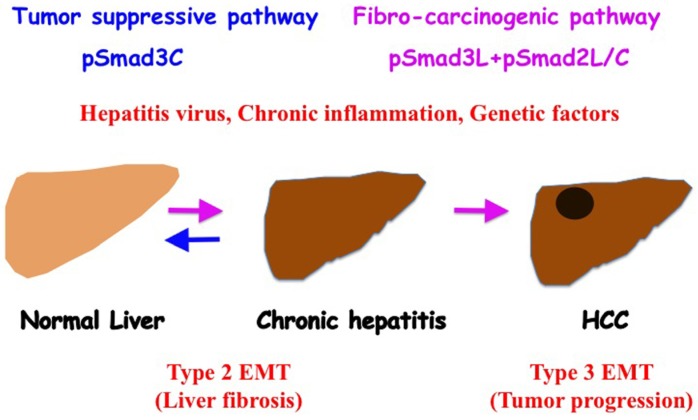
Phosphorylated Smad2/3 signaling fibro-carcinogenesis. As human hepatitis virus-related chronic liver diseases progress, chronic inflammation and hepatitis virus additively shift hepatocytic Smad phospho-isoform signaling from tumor-suppressive pSmad3C to the fibro-carcinogenic pSmad3L and pSmad2L/C pathway. Anti-viral therapy can reverse phospho-Smad signaling from fibro-carcinogenesis to tumor suppression. Type 2 EMT promotes liver fibrosis induced by chronic inflammation. Type 3 EMT exacerbates the HCC phenotype by upregulating invasive and metastatic potential.

Recent evidence suggests injured hepatocytes undergo EMT, and some of these epithelia-derived mesenchymal cells, however, may be capable of undergoing subsequent MET [38]. We have shown that HCV and HBV clearance restores human hepatocytic phospho-Smad signaling from fibro-carcinogenic pSmad3L (Ser-213) to the tumor-suppressive pSmad3C signaling in the early stage of chronic HCV or HBV of the patients [121,122]. Liver fibrosis regressed in most patients with mild liver fibrosis who achieved sustained virological response (SVR), but not in patients with advanced fibrosis (Figure 3). Hepatocytes maintain high carcinogenic pSmad3L (Ser-213) and cannot return to tumor-suppressive pSmad3C signaling, even after HCV clearance. Since genetic and epigenetic alterations of the major oncogenic pathway may lead to sustained linker phosphorylation of Smad3. Thereafter, pSmad3L (Ser-213) can transmit fibro-carcinogenic signals even after chronic inflammation resolves. In this situation, HCV clearance cannot reverse acquired fibro-carcinogenic Smad signaling.

## 7. Conclusions

We have provided a comprehensive overview of recently-reported clinical and basic research in the fibro-carcinogenesis of liver. Especially, we have focused on EMT and the TGF-β/Smad phospho-isoform in hepatitis virus-related liver diseases. A detailed understanding of the molecular mechanisms involved in progression to HCC is of fundamental importance in guiding the development of effective prevention and treatment for HCC.

Because HCC is a highly chemoresistant cancer, no effective systemic cytotoxic chemotherapy has been established [123]. Surgical resection or percutaneous intervention (radiofrequency ablation and ethanol injection) therapy is effective only at an early stage of HCC. Approximately 70% of these patients develop recurrent tumors within five years [124]. Transarterial chemoembolization is reserved for patient intermediate stage HCC without portal invasion or extrahepatic metastasis. Molecular target therapy, especially that targeting the angiogenesis pathway, is now developing as a novel anti-HCC therapy. Sorafenib is anti-angiogenic tyrosine kinase inhibitor; however, to date, none of these novel anti-angiogenic agents have exhibited superior efficacy to sorafenib. Although sorafenib is the only currently available therapeutic option for patients with advanced-stage HCC, they are required to have Performance Status 0–2 and Child-Pugh A [125].

Several kinds of clinical trials are testing novel molecular targets and other agents in the treatment of HCC. Cancer cell-directed oncogenic signaling pathways for advanced HCC treatment, including agents targeting the endothelial growth factor receptor (EGFR), the fibroblast growth factor receptor (FGFR), PI3K/Aki/mTOR, TGF-β, c-Met, insulin-like growth factor (IGF) signaling and histone deacetylase, have been actively explored. However, they did not prolong overall survival [126]. Such chemoresistance of HCC may relate to EMT-induced stem cell-like features. Some studies have shown that sorafenib inhibits β-catenin/the JNK pathway in liver cancer stem cells and mice bearing HepG2 cell-derived tumors [127,128,129]. Compounds that potently interfere with JNK-Smad signaling may be a new therapeutic target for HCC, and Smad phospho-isoform signaling can be a useful predictive biomarker for early assessment of pharmacologic interventions intended to suppress human fibro-carcinogenesis of the liver.
